# Meal Timing and Glycemic Control during Pregnancy—Is There a Link?

**DOI:** 10.3390/nu13103379

**Published:** 2021-09-26

**Authors:** Shengjie Zhu, Prasanth Surampudi, Nancy T. Field, Maria Chondronikola

**Affiliations:** 1 Department of Nutrition, University of California Davis, One Shields Ave, Davis, CA 95616, USA; hozhu@ucdavis.edu; 2 Department of Internal Medicine, Division of Endocrinology, Diabetes, and Metabolism, University of California Davis, 2315 Stockton Blvd, Sacramento, CA 95817, USA; psurampudi@ucdavis.edu; 3 Department of Obstetrics and Gynecology, University of California Davis, 2315 Stockton Blvd, Sacramento, CA 95817, USA; ntfield@ucdavis.edu; 4 Department of Nutritional Sciences and Dietetics, Harokopio University of Athens, El. Venizelou 70, 176 71 Kallithea, Greece

**Keywords:** glucose metabolism, time-restricted eating, intermittent fasting, gestation, gestation diabetes mellitus, hormones

## Abstract

Hyperglycemia during pregnancy and gestational diabetes mellitus (GDM) constitute an important public health problem due to their prevalence and long-term health consequences both for the mother and offspring. Results from studies in rodents and some clinical investigations suggest that meal time manipulation may be a potential lifestyle approach against conditions involving perturbations in glucose homeostasis (e.g., hyperglycemia, insulin resistance, diabetes, etc.). The purpose of this review is to summarize and critically evaluate the current literature on the role of meal timing and daily nutrient distribution on glycemic control during pregnancy. Only a small number of mostly observational studies have assessed the role of meal timing in glucose homeostasis during pregnancy. Food consumption earlier in the day and short-term fasting with adequate nutrient intake may improve glycemic control during the second and third trimester of gestation. Considering that the field of chrononutrition is still in its infancy and many questions remain unanswered, future prospective and carefully designed studies are needed to better understand the role of meal timing in metabolic homeostasis and maternal and fetal health outcomes during pregnancy.

## 1. Introduction

Gestational diabetes mellitus (GDM) and hyperglycemia during pregnancy are important public health problems due to their prevalence and long-term consequences both for maternal and child health. GDM affects approximately one in six births worldwide with the majority of the GDM cases reported in low- and middle-income countries [1]. The prevalence of GDM has almost doubled from 2006 (4.6%) to 2016 (8.2%) in the United States, while it appears to disproportionally affect low-income and minority populations [2].

Diagnosis of GDM can be confirmed using an one-step or two-step strategy at 24–28 weeks of gestation [3]. For the one-step strategy, a single elevated value of fasting plasma glucose (FPG) ≥ 92 mg/dL, 1 h post 75 g oral glucose tolerance test (OGTT) ≥ 180 mg/dL or 2 h post 75 g OGTT ≥ 153 mg/dL is consistent with the diagnosis of GDM. The two-step strategy includes a 1 h 50 g glucose load test (GLT) followed by a 100 g OGTT for those who screen positive (plasma glucose levels ≥ 130, 135, or 140 mg/dL 1 h post 50 g GLT). Using the two-step strategy, the diagnosis of GDM can be established when two or more of the following criteria are met during a 100 g OGTT: FPG ≥ 95 mg/dL, 1 h post 100 g OGTT ≥ 180 mg/dL, 2 h post 100 g OGTT ≥ 155 mg/dL, 3 h post 100 g OGTT ≥ 140 mg/dL.

Hyperglycemia during gestation and GDM have been tied to complications during pregnancy and the development of cardiometabolic disease for the mother and offspring. Specifically, hyperglycemia during pregnancy has been associated with increased odds for gestational hypertension, pre-eclampsia, and premature delivery [4,5,6,7]. Moreover, fetuses exposed to hyperglycemia in utero have a three-fold higher probability of fetal macrosomia [8], which is associated with increased odds of labor abnormalities, shoulder dystocia, and cesarean section [9]. After birth, infants born by mothers with GDM are more likely to experience hypoglycemia due to the existing hyperinsulinemia and hyperglycemia in utero [10,11]. Women with past medical history of GDM are at risk for the development of type 2 diabetes, hypertension, and cardiovascular disease during the first decade postpartum [12,13,14]. Similarly, offspring exposed to hyperglycemia during pregnancy are at high risk of development of the same chronic diseases later in life, which may be due to hyperglycemia-induced epigenetic changes [15].

The management of GDM and hyperglycemia during pregnancy involves lifestyle modifications with or without administration of glucose-lowering medications [16]. Adequate nutrition to promote fetal development and optimal weight gain, decreased simple carbohydrate intake, frequent self-blood glucose monitoring, and regular physical activity are the first-line treatment approach against hyperglycemia during pregnancy and GDM [17]. However, adopting behavioral modifications in a short period of time (even with adequate support) can be challenging [18]. Some patients may also require the administration of glucose-lowering agents to achieve glycemic control. Insulin administration is the primary pharmacological approach for the management of GDM [16]. Although insulin administration is very efficacious in the management of hyperglycemia, it requires carbohydrate counting with each meal to match insulin administration to the carbohydrate intake to achieve glycemic control. Glyburide and metformin have been also approved for the treatment of GDM by the US Food and Drug Administration [16]. However, their use is not recommended as a first-line pharmacological approach for the treatment of GDM, as their metabolites can cross the placenta, and their long-term safety remains to be determined [16]. Considering the limitations of the current modalities for the management of hyperglycemia during pregnancy, further research is needed to establish efficacious and safe approaches to improve glycemic control during pregnancy.

Over the last decade, there has been intense scientific interest to better understand the role of meal timing in metabolic health. Evidence from studies in rodents support that the synchronization of meal timing to the endogenous circadian rhythms regulating the efficiency of mechanisms involved in nutrient metabolism can improve metabolic health [19,20]. Recent findings support that restricting the daily eating window and/or shifting the timing of dietary intake earlier in the day may lead to improved glycemic control and insulin sensitivity in the non-pregnant state [21,22,23,24,25]. Further evidence supports that energy and/or carbohydrate distribution throughout the day and eating frequency may also affect glycemic control [26,27,28]. Therefore, synchronizing food intake to the endogenous timekeeping mechanisms affecting metabolic efficiency could conceivably improve maternal metabolic health and pregnancy outcomes. The purpose of this review is to summarize and critically evaluate the current literature on the interrelationship between meal timing, frequency, 24 h energy, and carbohydrate distribution on glucose homeostasis during pregnancy.

## 2. Endocrine Mechanisms Underlying the Alterations in Glucose Metabolism during Pregnancy

**Figure 1:**
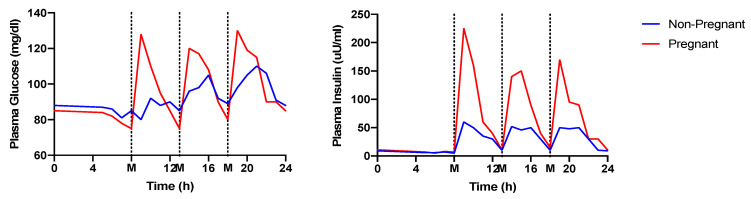
Diurnal circulating concentrations of glucose (left panel) and insulin (right panel) in healthy non-pregnant and healthy pregnant females during the third trimester of gestation. Adapted from reference [30].

**Figure 2:**
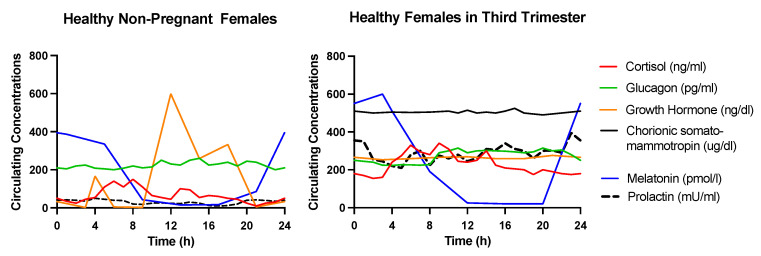
Circulating concentrations of glucoregulatory hormones in non-pregnant healthy females and healthy pregnant females during the third trimester. The data reported in references [32,33,34,35,36] were compiled to create this figure.

Pregnancy constitutes a state during which numerous physiological adaptations take place to promote fetal development. Glucose availability in the circulation increases, especially in the late second and third trimester to (in principle) ensure adequate fuel supply for the developing fetus [29]. Changes in the secretion of several glucoregulatory hormones (e.g., growth hormone (GH), human chorionic somatomammotropin (hCS), cortisol, glucagon, estrogen, progesterone, melatonin, and prolactin) contribute to the altered glucose homeostasis during pregnancy. However, this physiological response can sometimes lead to chronic hyperglycemia. To accommodate the higher circulating glucose concentration, postprandial insulin secretion also increases (Figure 1) [30]. Insufficient insulin secretion due to β-cell dysfunction and increased insulin resistance leads to hyperglycemia during pregnancy and GDM [31]. The section below summarizes the changes in the circulating levels of the major glucoregulatory hormones during healthy pregnancy and their effect on glucose metabolism. Figure 2 provides an overview of the trends in the 24 h circulating concentrations of the major glucoregulatory hormones in healthy non-pregnant females and during the third trimester of healthy gestation [32,33,34,35,36].

### 2.1. Growth Hormone (GH)

One of the important roles of GH is to ensure nutrient availability for the fetus. The circulating levels of GH follow a diurnal pattern in the non-pregnant state and in the first 15–20 weeks of pregnancy with the pituitary GH to constitute its main circulating form. Later in pregnancy, non-pulsatile placental GH becomes the predominant GH form in maternal circulation, while the pulsatile pituitary secretion decreases and pituitary GH becomes undetectable [37]. The circulating levels of GH are no longer rhythmic during the second and third trimester of pregnancy [33,38]. Apart from its role in fetal development, GH can directly or indirectly affect glycemic control. Elevated GH can contribute to hyperglycemia through multiple mechanisms, including decreased insulin-stimulated glucose uptake, increased endogenous glucose production, and altered glycogen synthesis. Briefly, GH is thought to stimulate glycogenolysis and potentially gluconeogenesis in the liver, leading to increased hepatic glucose production [39]. The binding of GH in the liver also stimulates the secretion of insulin-like growth factor 1 (IGF-1), which can bind to insulin receptors, leading to decreased insulin sensitivity [37]. Furthermore, GH stimulates adipose tissue lipolysis, increasing plasma free fatty acids availability, which can impair insulin-stimulated glucose disposal [39].

### 2.2. Human Chorionic Somatomammotropin (hCS)

Apart from its role in facilitating the exchange of nutrients and waste between mother and fetus, the placenta also secretes hCS, which has profound effects on maternal metabolism. hCS leads to impaired insulin sensitivity and glucose clearance, promotes adipose tissue lipolysis, and increases the availability of circulating amino acids [40]. As with GH, elevations in hCS can contribute to elevated maternal plasma IGF-1 concentrations and subsequently impact glucose metabolism [37]. Additionally, hCS increases serotonin in pancreatic islets, promoting β-cell expansion and proliferation [41]. hCS becomes detectable in maternal circulation by 3–4 weeks of gestation [42], and it peaks at 34 weeks of gestation, remaining high until delivery [43]. It does not follow a diurnal rhythm nor do its concentrations change in response to eating [34].

### 2.3. Prolactin

Prolactin is primarily synthesized by the anterior maternal pituitary gland, the uterus, and the fetal pituitary gland [44]. During gestation, prolactin stimulates implantation, placental development, and fetal growth. Its receptors are also expressed in various tissues, including those in pancreatic islets [45]. High prolactin exposure promotes β-cell replication, insulin secretion, and hyperinsulinemia, while it is also associated with impaired glucose tolerance and insulin resistance [46]. The circulating levels of prolactin increase with pregnancy, while they further increase after meals and in the night [32].

### 2.4. Cortisol

Cortisol is a catabolic hormone with glucoregulatory function secreted by the adrenal gland. Circulating cortisol concentration increases during late pregnancy and follows a diurnal pattern similar to the non-pregnant state [32,47]. The rising cortisol levels can influence plasma glucose through multiple mechanisms including increased maternal hepatic glucose production, altered β-cell function, and increased insulin resistance. Specifically, cortisol stimulates gluconeogenesis in the liver, increasing hepatic glucose production [48,49]. Furthermore, it decreases basal and insulin-stimulated glucose uptake in skeletal muscle and omental adipose tissue [50,51,52] and increases insulin secretion [53,54].

### 2.5. Glucagon

Glucagon is a catabolic hormone secreted by the α-cells of the pancreas and has pronounced effects on glucose metabolism. Activation of the glucagon receptor in liver stimulates glycogen breakdown, inhibits glycogen synthesis, and enhances hepatic glucose production [55,56]. Glucagon also decreases muscle glucose uptake, stimulates adipose tissue lipolysis, and decreases insulin secretion [55,56]. The multiple actions of glucagon can result in elevated blood glucose levels. Moreover, glucagon response to insulin-induced hypoglycemia is suppressed during pregnancy potentially due to decreased pancreatic islet sensitivity to the action of glucagon [32,57]. Glucagon circulating levels increase during late pregnancy and follow a similar circadian rhythm to that of the non-pregnant state [32].

### 2.6. Melatonin

Melatonin is secreted by the pineal gland and affects circadian rhythmicity and metabolic function in peripheral tissues [58]. Not only does it regulate insulin synthesis and secretion, but it also affects glucose clearance by regulating the expression of the GLUT4 glucose transporter and/or stimulation of the insulin signaling pathway [58]. Maternal melatonin levels increase during gestation primarily due to a pronounced increase in the amplitude of the melatonin peak at night with no phase shift [35,36,59].

In summary, current evidence supports that pregnancy is associated with profound changes in the levels of glucoregulatory hormones contributing to changes in glycemic control, insulin sensitivity, and insulin secretion. The majority of the glucoregulatory hormones (e.g., prolactin, cortisol, glucagon, melatonin) maintain their pre-pregnancy circadian/diurnal rhythmicity, while others (e.g., GH) become non-rhythmic. The synchronization of meal timing with the endogenous time-keeping mechanisms regulating metabolic efficiency may constitute a promising nutritional approach for the prevention and treatment of hyperglycemia during pregnancy.

## 3. Fasting Duration and Glycemic Control during Gestation

Over the last decade, there has been intense scientific interest in the role of fasting in metabolic health. Short-term fasting without starvation has been associated with improved glucose metabolism in the non-pregnant state [20,25]. Although the mechanisms underlying the metabolic benefits of fasting are not yet fully understood, they are thought to include a switch in substrate utilization (from glucose to lipids) and cellular stress resistance (reviewed in detail in refs. [60,61]). Pregnancy constitutes a distinct physiologic state; thus, results from studies in non-pregnant adults may not directly translate to the pregnant state. The role of fasting duration in glucose homeostasis during pregnancy remains largely unknown, and only a few studies have attempted to address this question.

Ramadan is a religious fasting event that involves abstinence from caloric intake from sunrise to sunset. The authors of a recent prospective observational study reported that Ramadan fasting (≈14 h daytime fasting) during the second trimester of pregnancy was independently associated with lower odds of GDM [62]. Furthermore, participants with a high number of fasting days during Ramadan (21–29 days) experienced lower weight gain (≈0.5 kg) compared to those that did not fast or fasted for 20 days or less. Ramadan fasting was not associated with increased odds for adverse pregnancy and fetal outcomes.

Another recent cross-sectional study in Singapore investigated the role of fasting duration on glycemic control (assessed by an OGTT after an 8–10 h overnight fast) during the end of the second trimester of gestation [63]. Eleven to twelve hours of overnight fasting were independently associated with lower FPG concentrations (≈2 mg/dL) compared to 9.1–10.9 h of overnight fasting. There was no association between overnight fasting duration and the 2 h post 75 g OGTT plasma glucose concentration.

The results of these studies provide preliminary evidence supporting the notion that short-term fasting without starvation may have metabolic benefits on glycemic control during pregnancy. However, the methodological limitations of those investigations (including their observational nature, self-reported data, and lack of comprehensive metabolic testing) underscore the need for further research aiming to better comprehend the effect of fasting on glucose metabolism and overall metabolic health during pregnancy. Considering that fasting beyond 14 h may lead to a state of “accelerated starvation” [64] during pregnancy, prolonged fasting should be likely avoided. Further research is needed to determine what is a safe fasting duration during pregnancy.

## 4. Meal Timing and Glycemic Control during Gestation

Recent evidence in non-pregnant adults supports that meal timing manipulation is a promising lifestyle intervention against obesity and its related metabolic complications (23, 24). However, the role of meal timing in metabolic health during pregnancy remains poorly understood.

Sacks et al. [65] conducted a randomized crossover trial in patients with GDM. The participants were offered test meals either in the morning (07:00) or at night (21:00). The two test meals were closely matched for energy but not for macronutrient composition. Despite its lower carbohydrate content (44 vs. 51%), the night test meal led to a higher postprandial glycemic response at 3 to 9 h compared to the morning meal test. Consistent with these results, Peterson et al. [66] reported that the correlation between carbohydrate intake and the 1 h postprandial glucose concentration was the strongest for dinner followed by lunch and breakfast.

Another more recent study investigated the role of meal timing on metabolic health in pregnant women with or without night eating syndrome (NES) symptoms during the third trimester of gestation [67]. NES is an eating disorder that presents with nocturnal hyperphagia or awakening for food intake, insomnia, and morning anorexia [68]. NES symptoms were assessed using a validated questionnaire. NES was associated with higher fasting plasma insulin concentration and the HOMA-IR (Homeostatic Model Assessment for Insulin Resistance) index (calculated using the following formula: FPG (mmol/L) times fasting serum insulin (mU/L) divided by 22.5). Moreover, a higher NES score was correlated with higher fasting insulin, hemoglobin A1c, and high-density lipoprotein cholesterol.

Taken together, these results suggest that meal timing and specifically eating in the night may affect metabolic health during pregnancy. Future prospective studies involving manipulation of meal timing are needed to determine its role in glucose homeostasis and insulin sensitivity during pregnancy.

## 5. Twenty-Four-Hour Energy and Carbohydrate Distribution and Glycemic Control during Gestation

Apart from the effect of meal timing and fasting duration on metabolic health, accumulating evidence supports that the distribution of energy and carbohydrate during the day may have important metabolic implications [26,27,69,70,71]. However, the role of energy and carbohydrate distribution throughout the day on metabolic health during pregnancy remains unclear, and only a small number of studies have investigated this relationship.

Chandler-Laney et al. conducted a cross sectional study to investigate the link between late-night energy and carbohydrate intake on glucose tolerance in healthy African American females in the third trimester of pregnancy [72]. Nighttime (20:00–05:59) energy intake was associated with lower dynamic β-cell response assessed by using a 2 h, 75 g OGTT in participants with obesity but not those with normal weight. In a larger cross-sectional study conducted in Singapore in predominantly Asian women during the second trimester of pregnancy, consumption of 50% or more daily energy intake at night (19:00–06:59) was independently associated with higher fasting glucose in lean participants [73] but not in participants with excessive adiposity (body mass index ≥23.0). Additionally, there was no association between postprandial glucose tolerance and daily energy distribution. The discrepancy in the reported results of those studies with regard to the modification effect of adiposity requires further investigation.

To further examine the link between 24 h energy/carbohydrate distribution, Rasmussen et al. performed a randomized crossover trial in normal weight patients with GDM. The participants followed a 4-day diet with high carbohydrate intake in the morning (50% daily energy/carbohydrate in breakfast and morning snack) and 4-day diet with high carbohydrate intake in the evening (50% daily energy energy/carbohydrate in dinner and night snack) [74]. High energy/carbohydrate intake in the morning led to lower FPG (83 vs. 92 mg/dL) compared to high energy and carbohydrate intake in the evening. Although having high energy and carbohydrate intake in the morning also improved the HOMA-IR index of insulin sensitivity, there were no significant differences between the two diets. The improvements in glycemic control with high energy and carbohydrate intake in the morning were also accompanied by an increase in mean glucose excursion and variability. These findings require further investigation as the small differences in the macronutrient intake between the two experimental conditions may confound the reported findings.

Current evidence supports that higher energy and/or carbohydrate intake later in the day may be linked to perturbed glucose homeostasis during pregnancy. Considering the limitations of the currently reported results (including cross-sectional study design or very short-term intervention with differences in total macronutrient composition and small number of participants), further research is needed to better understand the effect of the daily energy and macronutrient distribution in metabolic health during gestation.

## 6. Eating Frequency and Glycemic Control during Gestation

The role of meal frequency in metabolic health has been an issue of scientific debate for several decades. Although a number of studies support that eating frequency may play a role in body weight regulation and metabolic health [75,76,77], others do not [78,79,80,81]. The relationship between meal frequency and metabolic health during pregnancy remains poorly understood. Results from a cross-sectional study indicate that the number of daily eating occasions is independently associated with higher 2 h postprandial glucose after 75 g OGTT in normal-weight females in the second trimester of gestation [63]. Eating frequency was not associated with fasting plasma glucose concentrations.

## 7. Future Research

Current evidence (predominantly from observational studies and small short-term clinical investigations—summarized in Table 1) supports that meal timing may affect glycemic control during pregnancy. However, results from observational studies cannot be used to establish causation. Therefore, well-controlled intervention studies including detailed metabolic and other clinical outcomes are needed to comprehensively investigate the role of meal timing, frequency, and 24 h energy and macronutrient distribution in glucose homeostasis and metabolic health particularly in target populations at high risk for the development of metabolic disease. Once the answers to these questions have been clarified, it will be important to understand how this knowledge can be translated into clinical practice to safely and effectively improve maternal and neonatal health outcomes.

## 8. Conclusions

GDM and hyperglycemia during pregnancy affect a large percentage of women. In addition to the role of nutrition quantity and quality in metabolic health and pregnancy outcomes, it is also critical to understand the role of meal timing. Overall, the current literature supports that meal timing, frequency, and 24 h energy and macronutrient distribution may play a role in the regulation of metabolic health during gestation. However, important methodological limitations of the prior studies limit their ability to conclusively establish the role of meal timing in glycemic control during pregnancy. Further research is needed to determine the role of meal timing, frequency, daily energy, and macronutrient distribution on maternal and fetal metabolic regulation and outcomes. Future investigations will also help establish safe, efficacious, and equitable chrono-nutrition lifestyle interventions to improve the health of the population and generations to come.

## Figures and Tables

**Table 1:** Summary table of studies on meal timing and glucose metabolism during pregnancy.

Author, Year, Study Design	Population	Methods	CHO and EI Distribution, and Appetite	Metabolic Outcomes	Other Outcomes
Peterson et al., 1991 [66] Cross-sectional study	Females with OW and GDM in 3rd trimester (*n* = 14)	Dietary advice to consume 24 kcal/kg/d. Self-prepared 3 meals and 3 snacks per day. Energy distribution: B: 12.5%, L: 28%, D: 28%, two S: 10.5% each.	NA	↑ correlation between CHO and 1 h pp glucose during dinner	NA
Sacks et al., 1999 [65] 1-Day randomized crossover trial	Females in 3rd trimester singleton with GDM (*n* = 16 OB, 14 NW; *n* = 23, 75% Hispanic)	9 h mixed meal test after ≥5 h fasting AM meal test (07:00): 564 kcal, 51% CHO, 17% protein, 32% fat PM meal test (21:00): 567 kcal, 44% CHO, 26% protein, 30% fat	NA	AM vs. PM meal test ↑ 1 h pp glucose and insulin ↑ glucose decline rate Ø 2–9 h pp insulin, pp insulin/glucose ratio ↓ FFA 2–3 h and ↑ 5–9 h	NA
Chandler-Laney et al., 2016 [72] Cross-sectional	Healthy African American females in 3rd trimester singleton pregnancy (*n* = 20 NW, *n* = 20 OB)	3-day food record verified in-person interview OGTT after >12 h overnight fast Daytime intake: 06:00–19:59 Nighttime intake: 20:00–05:59	OB vs. NW Ø earliest and latest EI time Ø EI after 20:00 ↑ daily EI (640 kcal) trend for ↑ nighttime CHO% intake Ø total daily macronutrient composition	Both groups - Nighttime EI associated with ↓ dynamic β-cell response NW group: no association with metabolic parameters OB group: - Nighttime EI associated with ↓ dynamic β-cell response	NA
Loy et al., 2016 [73] Cross-sectional	Asian females in late 2nd trimester pregnancy (*n* = 534 NW, *n* = 451 OW)	Nutrition habits obtained using 24 h food recall. OGTT after overnight fast AM eater (*n* = 83): >50% EI 07:00–18:59 PM eater (*n* = 147): >50% EI 19:00–06:59	PM vs. AM eating NW: ↓ total EI OW: ↓ %CHO trend for ↑ %fat	NW: PM eating associated with ↑ fasting glucose (2.5 mg/dL), but not 2 h OGTT glucose OW: PM eating not associated with fasting or 2 h OGTT glucose	Ø PA Ø Sleep duration
Loy et al., 2017 [63] Cross-sectional	Asian females in late 2nd trimester singleton pregnancy (*n* = 1061)	Nutrition habits obtained using 24 h food recall and 3-day food record. OGTT after 8–10 h overnight fast Night fasting: hours with no EI 19:00–06:59 EO: EI ≥50 kcal with ≥15 min apart	Participants with ↑ night fasting had: ↓ daily EI and EO - ↑EI% after 1900 h and %protein Participants with ↑daily EO had: - ↑ daily EI - ↓ %protein - ↓ nighttime fasting	Night fasting associated with: ↓ fasting glucose (9.1–10.9 vs. 11–12 h/day) Ø 2 h OGTT glucose Daily EO associated with: Ø fasting glucose ↑ 2 h pp glucose (4 vs. 5–10 EO/day)	Participants with ↑ night fasting: - ↑ BMI in early 1st trimester - Sleeping earlier Participants with ↑ EO had ↓ BMI in early 1st trimester and PA
Deniz et al., 2019 [67] Cross-sectional study	OW females in 3rd trimester healthy singleton pregnancy (*n* = 17 NES, *n* = 131 non-NES)	NES score assessed with validated questionnaire Hunger assessed via interview Infant information obtained by medical record.	NES vs. Non-NES ↑ morning hunger, Ø night hunger ↑ B skipping	NES vs. Non-NES ↑ fasting insulin, HOMA-IR index, and HDL-c trend for ↑ fasting glucose and total cholesterol Ø BMI, LDL-c, HbA1c NES score correlated with fasting insulin, HOMA-IR, HbA1c, HDL-c	NES vs. Non-NES trend for ↑ fetal weight
Safari et al., 2019 [62] Prospective observational study	Females in 2nd trimester healthy pregnancy	All data collected via questionnaires and hospital records	NA	Ramadan Fasting vs. No fasting ↓ GDM prevalence	Ramadan Fasting vs. No fasting ↓ GWG (~0.5 kg) Ø pre-eclampsia, preterm labor, fetal birth weight or other fetal outcomes
Rasmussen et al., 2020 [74] 4-Day randomized crossover trial	NW females in 3rd trimester with GDM (*n* = 12)	Eucaloric meal intervention (46% CHO, 20% protein, 34% fat). Self-prepared 3 meals and 2 snacks per day High CHO and EI AM - Energy distribution: B: 25–30%, MS: 15–20%, L: 25–30%, AS: 10–15%, D: 15–20% - CHO distribution: AM: 50% (B: 30–35%, MS; 15–20%), afternoon: 40% (L: 25–30%, AS: 10–15%), D: 10% High CHO and EI PM - Energy distribution: B: 15–20%, L: 25–30%, AS: 10–15%, D: 30–35%, NS: 15–20% - CHO distribution: B 10%, afternoon: 40% (L: 25–30%, AS: 10–15%), D 50% (D 30–35%, NS 15–20%)	High CHO and EI AM vs. PM Ø daily EI, CHO and protein intake ↓ fat intake (9 g)	High CHO and EI AM vs. PM ↓ 4-day mean glucose (5.4 mg/dL) ↓ day 4 fasting glucose (9.0 mg/dL) ↑ mean glucose excursion (10.8 mg/dL) ↑ variation coefficient (5.2%) Ø change in insulin sensitivity, total, LDL-c, HDL-c, TG	NA

All data are collected from females with no insulin or medication treatment. AM: morning; AS: afternoon snack; AUC: area under the curve; B: breakfast; BMI: body mass index; CHO: carbohydrate; D: dinner; DI: disposition index; EI: energy intake; EO: eating occasion; FFA: free fatty acids; GDM: gestational diabetes mellitus; GWG: gestational weight gain; HbA1c: hemoglobin A1c; HDL-c: high-density lipoprotein cholesterol; HOMA-IR: homeostatic model assessment for insulin resistance; L: lunch; LDL-c: low-density lipoprotein cholesterol; MS: morning snack; NA: not applicable; NES: night eating syndrome; NS: nighttime snack; NW: normal weight; OB: obesity; OGTT: oral glucose tolerance test; OW: overweight; PA: physical activity; PM: afternoon; pp: postprandial; S: snack; TG: triglyceride; Ø: no significant difference; ↑: higher; ↓: lower.

## Data Availability

Not applicable.
